# Multilayer Films Based on Chitosan/Pectin Polyelectrolyte Complexes as Novel Platforms for Buccal Administration of Clotrimazole

**DOI:** 10.3390/pharmaceutics13101588

**Published:** 2021-09-30

**Authors:** Joanna Potaś, Emilia Szymańska, Magdalena Wróblewska, Izabela Kurowska, Mateusz Maciejczyk, Anna Basa, Eliza Wolska, Agnieszka Zofia Wilczewska, Katarzyna Winnicka

**Affiliations:** 1 Department of Pharmaceutical Technology, Faculty of Pharmacy, Medical University of Białystok, 15-222 Białystok, Poland; joanna.potas@umb.edu.pl (J.P.); esz@umb.edu.pl (E.S.); magdalena.wroblewska@umb.edu.pl (M.W.); 2 Department of Polymers and Organic Synthesis, Faculty of Chemistry, University of Białystok, 15-245 Białystok, Poland; i.kurowska@uwb.edu.pl (I.K.); agawilcz@uwb.edu.pl (A.Z.W.); 3 Doctoral School of Exact and Natural Sciences, University of Białystok, 15-245 Białystok, Poland; 4 Department of Hygiene, Epidemiology and Ergonomics, Faculty of Health Sciences, Medical University of Białystok, 15-222 Białystok, Poland; mateusz.maciejczyk@umb.edu.pl; 5 Department of Physical Chemistry, Faculty of Chemistry, University of Białystok, 15-245 Białystok, Poland; abasa@uwb.edu.pl; 6 Department of Pharmaceutical Technology, Faculty of Pharmacy, Medical University of Gdańsk, 80-416 Gdańsk, Poland; eliw@gumed.edu.pl

**Keywords:** multilayer film, polyelectrolyte complex, chitosan, pectin, clotrimazole, oral candidiasis, buccal delivery

## Abstract

Buccal films are recognized as easily applicable, microbiologically stable drug dosage forms with good retentivity at the mucosa intended for the therapy of oromucosal conditions, especially infectious diseases. Multilayer films composed of layers of oppositely charged polymers separated by ionically interacting polymeric chains creating polyelectrolyte complexes represent very interesting and relatively poorly explored area. We aimed to develop the antifungal multilayer systems composed of cationic chitosan and anionic pectin as potential platforms for controlled delivery of clotrimazole. The systems were pharmaceutically characterized with regard to inter alia their release kinetics under different pH conditions, physicomechanical, or mucoadhesion properties with using an animal model of the buccal mucosa. The antifungal activity against selected *Candida* sp. and potential cytotoxicity with regard to human gingival fibroblasts were also evaluated. Interactions between polyions were characterized with Fourier transform infrared spectroscopy. Different clotrimazole distribution in the films layers highly affected their in vitro dissolution profile. The designed films were recognized as intelligent pH-responsive systems with strong antifungal effect and satisfactory safety profile. As addition of chitosan resulted in the improved antifungal behavior of the drug, the potential utilization of the films in resistant cases of oral candidiasis might be worth of further exploration.

## 1. Introduction

Polyelectrolyte complexes (polycomplexes, PECs) are three dimensional structures created by the oppositely charged polyions combined by reversible electrostatic interactions and supported by hydrogen and/or hydrophobic forces [1]. While among cationic macromolecules used in the PECs preparation, chitosan (CS) and its derivatives are most commonly used, a broad spectrum of negatively charged polymers, including natural, semi–synthetic, and synthetic ones, have been incorporated into PECs–based systems so far [2]. CS as a natural-origin polysaccharide with multifunctional character which covers antibacterial, antifungal, antioxidant, anti-inflammatory, or hemostatic behavior has been gaining attention of pharmaceutical technologists for many years, even though issues related to its pH-dependent solubility, high susceptibility to the presence of ions or poor mechanical, and rheological properties have always been a challenge [3,4]. Complexation of CS amino groups with negatively charged polymers without addition of toxic cross-linking agents was recognized as an alternative for CS characteristics improvement with simultaneous maintaining its unique performance.

Oral candidiasis is an oromucosal fungal infection caused by pathogenic forms of *Candida* species with special focus on *C. albicans* responsible for ~95% cases of the disease. Among numerous factors which favor the transition of commensal *Candida* to pathogenic species, the coexisting oromucosal conditions, such as lichen planus, xerostomia, as well as these associated with insufficient oral hygiene, poorly fitting dentures, hormonal disorders (diabetes mellitus, Cushing syndrome), or local/systemic antibiotic and corticosteroid therapies should be indicated. Groups which are particularly affected by chronic and difficult-to-treat lesions with tendency toward systemic fungal infections are immunocompromised oncology and HIV infected patients [5]. Despite high interest in new antifungal substances exploration, polyenes (amphotericin B, nystatin), azoles, including imidazoles (clotrimazole, miconazole, ketoconazole), and triazoles (fluconazole, itraconazole, posaconazole, isavuconazole), as well as echinocandins (caspofungin, micafungin, anidulafungin) are main classes of antifungals still fundamental for the therapy of oral candidiasis [6]. Taking into consideration the importance of drug carriers in modulation of pharmacological effect of the incorporated active substances, exploration of novel platforms for the existing antifungal agents which might improve their antimicrobial effect, especially with regard to the resistant cases of candidiasis, should be undertaken.

Clotrimazole (CLO) is a practically water-insoluble (0.49 mg/mL [7]), broad-spectrum agent dedicated for the therapy of dermatophyte and *Candida* infections [8]. Because of the low bioavailability, it is recommended for the topical treatment of fungal infections of the oral cavity, vaginal mucosa, or the skin. As antimycotic properties of the drug were indicated in the late 1960s, the multitarget mechanism of CLO antifungal behavior is well known, although the ability of lanosterol–14–demethylase (CYP 51) inhibition which affects ergosterol production and then the fungal cell membrane function is regarded as the most important for its pharmacological effect. Besides concentration-related fungistatic or fungicidal activity of CLO, its antibacterial behavior against Streptococcus mutans, responsible for dental caries and complications that arise from this, has been recently observed [9]. Given the synergistic effect of both aforementioned pathogens in the development of oral cavity diseases, attention given to CLO and platforms which might improve its antimicrobial performance might be therefore beneficial for broadly understood oral health.

Buccal delivery of antimicrobials is a common practice in the therapy of oromucosal infections. Nevertheless, considering the character of the buccal mucosa, including constant flowing down of the saliva or the risk of accidental swallowing of the product administered, the necessity of highly mucoadhesive and well tolerable buccal systems development should be highlighted. Buccal films are recognized as easily applicable, microbiologically stable drug dosage forms with good retentivity at the mucosa. Over the last years, numerous scientific approaches dedicated to the development of buccal films as potential platforms for either local [10,11,12] or systemic [13,14,15] drugs have been undertaken. Apart from “traditional” monolayer films, systems with more complex structure have been designed as well, nevertheless, they are predominantly composed of layers of neutral or positive polymers dedicated for incorporation of different active substances or they are “equipped” with an additional layer of water-insoluble material ensuring unidirectional drug release at the application site [11,16]. Our attention is particularly drawn by multilayer systems composed of layers of oppositely charged polyelectrolytes separated by layers of interacting polymers chains creating PECs. Even though they have been mostly investigated for biomedical purposes as potential antimicrobial coatings for coronary stents, tissue engineering materials or pervaporation membranes used in the chemical technology [17,18], evaluation of their applicability in drug delivery might be worth of further exploration [19]. Responding to the limitations of the multilayer systems technology based on the layer-by-layer (Lbl) technique with using sophisticated and expensive automatic dipping machines or laboratory spinnerets, we focused on a simple, affordable, and easy-to-scale-up solvent evaporation technique and its potential utility in CS PECs multilayer films preparation. Shortly, the multilayer systems are mostly identified with the aforementioned Lbl method, which allows for the deposition of the oppositely charged components with nanoscale control over size, structure or shape of the final composite [20]. As different types of Lbl technique, including dipping, spraying, or spinning-assisted ones, have been developed so far, wide utilization of polyelectrolyte multilayers (PEMs) in coating geometrically diversified medical devices can be observed [21]. Nevertheless, despite the undeniable novelty of Lbl-assembled composites, high costs and low drug entrapment efficiency are listed as the main reasons of their limited use as drug carriers. Similarly, techniques of films preparation such as inkjet printing [22], fused deposition modeling (FDM), 3D printing [23], or electrospinning [24] seem to be promising, however still requiring very sophisticated equipment. Apart from time-consuming stages of drying, simplicity, and affordability of the solvent evaporation method are the reasons of its widespread use in the pharmaceutical technology, especially with regard to insoluble or unstable active substances (e.g., bioactive molecules, such as proteins or genetic material) for which providing an optimal drug loading can be particularly challenging.

High molecular weight (HMW) CS was selected as optimal for the films’ preparation as, according to Grimling et al. [25], its synergistic antifungal effect with CLO against *Candida* sp. was observed. In addition, the ability of CS to improve the dissolution rate and bioavailability of poorly soluble drugs has been observed [26,27]. Pectin (PC)—water-soluble natural polysaccharide composed of D–galacturonic acid monomers linked by α–(1, 4)–glycosidic bonds [28] with anionic carboxylic acid groups—was chosen as a negatively charged component of the developed PECs. Depending on the degree of methyl esterification, PC might be divided into high methoxy (≥50%) and low methoxy (<50%) type. In light of the available literature, combinations of CS with PC are regarded as highly beneficial for buccal/periodontal monolayer films preparation. Nevertheless, development and quality assessment of simply prepared multilayer systems can be treated as a novel practice toward CS/PC films technology. Polyethylene glycol 400 (PEG 400) was used to increase solubility of CLO, however, its effect on antibacterial or antioxidant properties as well as the enhanced water solubility and complexation ability of CS was also regarded as beneficial for the films’ preparation [29,30]. The films were pharmaceutically characterized with regard to inter alia their swelling behavior, drug release kinetics under different pH conditions, physicomechanical, or mucoadhesion properties. Furthermore, the antifungal activity against three selected *Candida* sp. and cytotoxic effect of the designed systems by using four independent tests were evaluated. To assess the interactions between CS, PC, and CLO, attenuated total reflectance Fourier transform infrared spectroscopy (ATR–FTIR) and differential scanning calorimetry (DSC) analyses were carried out. The internal structure of the films was picturized by using scanning electron microscopy (SEM).

## 2. Materials and Methods

### 2.1. Materials

High molecular weight CS derived from snow crabs (degree of deacetylation: 79.1%, viscosity of 1% solution in 1% acetic acid: 756 mPa·s, molecular weight: 200–500 kDa) was provided by Heppe Medical CS GmbH (Haale, Germany). High methoxy amidated PC from citrus peel (GENU^®^ pectin, USP–H type) was kindly obtained from CP Kelco (Atlanta, GA, USA) (degree of esterification: 65–75%, methoxy groups content: 6.7–12.0%, viscosity of 2% solution: 200–450 mPa·s, galacturonic acid content: ≥74%). CLO and glycerol were purchased from Fagron (Kraków, Poland). Disodium hydrogen phosphate, potassium dihydrogen phosphate, sodium hydroxide, and 85% lactic acid were purchased from Chempur (Piekary Śląskie, Poland). In turn, PEG 400 was obtained from Avantor Performance Materials Poland S. A. (Gliwice, Poland). Tween 80 and dimethyl sulfoxide (DMSO) were provided by Sigma Aldrich (St. Louis, MO, USA). Methanol for HPLC analysis was obtained from J. T. Baker (Phillipsburg, NJ, USA), while water for HPLC was distilled and passed through a reverse osmosis system Milli-Q Reagent Water System (Billerica, MA, USA). Stock cultures of *Candida albicans* ATCC^®^ 10231, *C. krusei* ATCC^®^ 6528, and *C.* *Parapsilosis* ATCC^®^ 22019 and Sabouraud’s dextrose agar were obtained from Biomaxima (Lublin, Poland). Sodium chloride (0.9%) was from Polpharma S. A. (Starogard Gdański, Poland). Nylon membrane filters (0.45 µm) were provided by Millipore (Billerica, MA, USA). Commercially available product with 10 mg/g CLO (Clotrimazolum GSK, GlaxoSmithKline Pharmaceuticals S. A., Poznań, Poland, series: FW2D, expiry date: 08.2023) was utilized as a reference in the antifungal test. Human gingival fibroblasts (PCS–201–018) were obtained from ATCC (Manassas, VA, USA). Fetal bovine serum (FBS), Dulbecco’s modified Eagle’s medium (DMEM), phosphate-buffered saline (PBS) pH 7.0, and trypsin were purchased from Gibco (Gaithersburg, MD, USA). Penicillin, streptomycin (solution stabilized with 10,000 units penicillin and 10 mg streptomycin per mL, 0.1 μm filtered, BioReagent, suitable for cell culture, cat. no P4333–100 mL), 3–(4,5–dimethylthiazol–2–yl)–2,5–diphenyltetrazolium bromide (MTT), glycine, sodium chloride, scintillation fluid Ultima Gold XR, H_2_O_2_, 2′,7′–dichlorofluorescein diacetate (DCFH–DA) and sodium dodecyl sulphate were purchased from Sigma-Aldrich (St. Louis, MO, USA or Steinheim, Germany). RealTime–Glo^TM^ Annexin V Apoptosis and Necrosis Assay Kit was obtained from Promega (Madison, WI, USA).

The simulated saliva solution (SS) composed of 0.1 M disodium hydrogen phosphate and 0.1 M potassium dihydrogen phosphate (according to Marques et al. with modifications [31]) was used for the research analyses.

Porcine buccal mucosa was obtained from the veterinary service of local slaughterhouse (Turośń Kościelna, Poland). The model tissue was dissected immediately after killing an animal, rinsed with saline and stored at −20 °C for three days. Defrosted model buccal mucosa was first rinsed with the SS and then subjected to the analyses as needed.

### 2.2. Preparation of Films

PC solution (2% (*w/w*); pH 3.4) was prepared by dissolving the high methoxy amidated polymer in water at room temperature with using a magnetic stirrer (Heidolph Instruments, Schwabach, Germany). The concentration of PC was chosen at the stage of preliminary studies, according to the previous reports dedicated to PC films [32,33] and our personal observations about the optimal viscosity of the polymer solution. CS solution (1% (*w/w*)) was obtained by dispersing the high molecular weight CS in 1% (*v/v*) lactic acid under gradual mixing on the magnetic stirrer at about 40 °C. To minimize potential irritation of the buccal mucosa, the pH of the CS solution was adjusted to 3.0 using 0.5 M NaOH. The polymers dispersions were prepared with addition of 0.25% (*w/w*) glycerol to improve the films plasticity. The applied polymers ratio was used with reference to the preliminary turbidimetric analysis, which indicated 1:2 weight ratio of CS to PC as optimal for the most intense stoichiometric PECs formation (data not presented in the article). The films were prepared by using simple layer-by-layer deposition of the polymers solutions on Petri dishes. For drug-loaded films F1–F3, different distribution of CLO in the films layers was applied (Table 1). The accurately weighted amount of CLO was solubilized in PEG 400 and then the required amount of PC solution was gradually added to the mortar with the drug and the solubilizer. Slow adding and careful mixing of the components aimed to obtain a homogenous drug dispersion. The prepared mixtures were subsequently casted on Petri dishes and stored in a fridge to remove air bubbles generated during the mixing process. They were subsequently dried in the oven at 30–35 °C for approximately 24 h for pre-gelation of the polymer casted. “Layer” of CS was prepared according to the above procedure and then carefully casted on the previous one (Table 1). Dried membranes were cut into 2 cm × 3 cm pieces with total CLO content 1.5 mg/cm^2^ [34], then wrapped individually with an aluminum foil and stored in a desiccator. As needed, films were divided into 1 cm^2^ or 4 cm^2^ pieces right before the analyzing procedure by using a guillotine for paper (Fellowes, Doncaster, UK). The placebo films (PF) were prepared as described above with addition of PEG 400.

### 2.3. Evaluation of Films Thickness and Weight Variations

The thickness of the systems was evaluated with a thickness gauge (Mitutoyo, Kawasaki, Japan) at three different points (upper, middle and lower part of 1 cm^2^ film) for at least three randomly chosen samples. To evaluate the films weight variations, the average weight of minimum three selected 1 cm^2^ systems was calculated.

### 2.4. pH Analysis

pH measurements were performed by using a glass electrode of the pH meter Orion 3 Star (Thermo Scientific, Waltham, MA, USA) at 25 ± 1 °C for the polymer solutions. In addition, the microenvironmental pH of the films with the surface area of 1 cm^2^ was measured after 4-hour swelling in 5 mL of SS pH 6.8, by placing the electrode in a beaker with a swollen formulation.

### 2.5. Moisture Content

Moisture content in 2 cm × 3 cm films was evaluated with moisture analyzer balance (Radwag WSP 50SX, Radom, Poland) at 80 °C.

### 2.6. High-Performance Liquid Chromatography (HPLC) Analysis of CLO

Quantitative analysis of CLO was performed by using HPLC system Agilent Technologies 1200 equipped with G1312A binary pump, G1316A thermostat, G1379B degasser, and G1315B diode array detector (Agilent, Waldbronn, Germany), by using Zorbax Eclipse XDB-C18, 4.6 × 150 mm, 5 µm column (Agilent Technologies, Waldbronn, Germany) maintained at 30 °C with a validated method. The mobile phase was composed of methanol and phosphate buffer pH 7.4 (80:20, *v/v*) under the flow rate of 1 mL/min. Detection of CLO was performed for 20 µL sample injected into HPLC system and analyzed at a wavelength of 210 nm, and at the retention time of 6.2 min [35]. For that purpose, the predetermined calibration curve (y = 151.08x − 15.058, R^2^ = 0.999) over the concentration range of 2.5 to 15.0 µg/mL was utilized. Analysis of the results was carried out with Chemstation 6.0 software.

### 2.7. Determination of Drug Content

Concentration of CLO incorporated into the films was determined for 1 cm^2^ films samples placed in 25 mL flasks with 10 mL of methanol:1% (*v/v*) lactic acid 4:1 (*v/v*) mixture. Protected with parafilm flasks were agitated at 150 rpm in the water bath (37.0 ± 0.5 °C) for 24 h. After that time, the contents of the flasks were filtered through 0.45 µm nylon filters, ten times diluted, and analyzed by the HPLC method described above (Section 2.6.).

### 2.8. Physicomechanical Analysis

Mechanical properties of 2 cm × 3 cm films were characterized by tear resistance (TR), tensile strength (σ_s_), percent of elongation at break (ε_s_), and Young’s (elastic) modulus (E) with using Texture Analyzer TA.XT. Plus (Stable Microsystems, Godalming, UK) at 25 ± 2 °C [11,36,37,38]. While tear resistance and tensile strength represent the stress needed for film breaking, percent of elongation at break describes the possible material deformation until it tears. Young’s modulus measures in turn material resistance to deformation and might be indicated by the slope on the stress/strain curve. With increasing the slope, Young’s modulus grows. Generally, for hard and brittle films, higher values of both Young’s modulus and tensile strength are noted [39,40].

The experimental parameters were set during the preliminary experiments with reference to the previous reports [40]. Based on the tension test mode, the grips were extended with pre-test, test, and post-test speed of 1 mm/s with a cell loading of 5 kg. Initial distance between the tensile grips was 20 mm.

The value of TR describes the force needed for tearing or rupturing a film, whereas σ_s_ can be calculated by using the following formula:

σ_s_ = F/S,

where F is the applied stress, S is the cross-section area of the film, and ε_s_ was determined by the following equation:

ε_s_ = [(l − l_0_)/l_0_] × 100,

where l_0_ is the initial length of the film, and l is the length of the film after elongation. As Young’s modulus quantifies the relationship between tensile stress (σ_s_) and axial strain (ε_s_), it was calculated as presented below,

E = F × l_0_/S × Δl,

where F is the applied stress, S is the cross-section area of the film, and Δl is the amount by which the length of the film changes [41]. Young’s modulus was calculated from a linear region of the stress/strain plot.

Furthermore, each film was evaluated in terms of flexural strength (folding endurance). For that purpose, the number of folds a film withstands before it breaks after exposure to constant load was determined [39,40,42].

### 2.9. Scanning Electron Microscopy (SEM)

The films were assessed morphologically with scanning electron microscope (Inspect™ S50, FEI Company, Hillsboro, OR, USA) at room temperature. For that purpose, pieces of the films were placed on adhesive tapes fixed to the surface of a special stand and gold sprayed. They were observed using different magnifications on two different planes.

### 2.10. Differential Scanning Calorimetry (DSC) Analysis

Thermal analysis of the films was conducted by using previously calibrated automatic thermal analyzer system (DSC TEQ2000, TA Instruments, New Castle, DE, USA) at a temperature range from 40 °C to 300 °C and a heating rate of 20 °C/min in argon atmosphere. 3 mg samples of F1–F3, placebo films, single polymers, CLO, and physical mixture of PC and CS (2:1, *w/w*) were analyzed in aluminum pans hermetically sealed with lids using an empty one as a reference.

### 2.11. Attenuated Total Reflectance Fourier Transform Infrared Spectroscopy (ATR–FTIR)

All spectra were collected using a Thermo Scientific Nicolet 6700 FTIR spectrophotometer (Waltham, MA, USA) equipped with diamond ATR. The spectra were compared to the background spectra, and 32 scans in the range between 500 cm^−1^ and 4000 cm^−1^ were taken. The spectra of each film were recorded from both sides.

### 2.12. Zeta Potential Measurement

Zeta potential of CLO particles was determined with Zetasizer NanoZS90 (Malvern Instruments, Malvern, UK) using Zetasizer Software 6.20. 5% (*w/w*) CLO suspensions in water and in the SS with addition of 0.5% (*w/w*) Tween 80 adjusted to pH 4.8 or 6.8 were prepared with using mortar and pestle, corresponding to the concentration of the antifungal in the films (about 5–7.5% (*w/w*), depending on the films weight). The addition of Tween 80 aimed to enhance CLO solubility. After one-hundred-fold dilution of the prepared dispersions with the suspending medium, zeta potential measurements were performed at 25 °C with equilibration time of 60 s. Each sample was analyzed three times directly after the suspension preparation and after 24 h.

### 2.13. In Vitro Release Study

In vitro release study was performed for films with surface area of 1 cm^2^, with USP dissolution apparatus type II (Erweka Paddle Dissolution tester Type DT 600HH, Heusenstamm, Germany) at 37.0 ± 0.5 °C. To obtain the sink conditions for CLO, 100 mL of the twenty-fold diluted SS with addition of 0.5% (*w/v*) Tween 80 was used. To evaluate the effect of pH on the release kinetics of the systems formed, the SS adjusted to pH 4.8 and 6.8, which mimics the oral cavity environment after and before eating, was used [31]. At the set time intervals, 1 mL samples of the release medium were withdrawn, then filtered through nylon filters with 0.45 nm pore size and analyzed by using the HPLC method which was described above (Section 2.6.). The analyses were repeated in triplicate.

### 2.14. Swelling and Disintegration Study

Performance of the films in the presence of SS was assessed with reference to our previously described method for CS/tragacanth/xanthan gum hydrogels, for buccal administration [43]. Given the variations in the drug release profiles observed for different pH conditions (Section 3.7.), the swelling test was conducted for films immersed in 15 mL of the SS adjusted to pH 4.8 and 6.8. 1 cm^2^ films were carefully weighted and placed in the baskets dedicated for USP Dissolution tests [44]. Twenty-five milliliter beakers with baskets were then filled with the swelling medium, protected with parafilm and thermostated on a water bath at 37.0 ± 0.5 °C. After 15, 30, 60, 90, 120, and 240 min, the baskets were removed from the beakers, carefully drained with cellulose wadding and then weighted using the analytical balance. Based on the obtained values of mass fluctuations, the degree of swelling (α) was calculated. For that purpose, the following equation was used:

α (%) = (W_s_ − W_0_)/W_0_ × 100,

where W_s_ is the weight of a film after swelling, and W_0_ is the initial weight of a film [45]. The study was performed in triplicate.

### 2.15. Erosion Test

The films were weighted and soaked in the SS pH 4.8 and 6.8 at 37.0 ± 0.5 °C. After 15 min and 60 min, the samples were removed from the media and dried in a hot air oven at 50 °C to obtain a constant weight (W_d_). Percentage of erosion was calculated by using the following formula:

Erosion (%) = (W_0_ − W_d_)/W_0_ × 100,

where W_0_ is the initial weight of a film, and W_d_ is the weight of a film after soaking in the SS [38].

### 2.16. Determination of Mucoadhesiveness

Because of the limited water solubility of CS, the polycation layer was indicated as gradually disintegrating reservoir of a drug which we have decided to put in contact with a mucous membrane. In turn, relatively fast eroding PC layer was found to provide initial dosage of the antifungal to the oral cavity and so the mucoadhesion of PC was not the object of the analysis.

Mucoadhesive properties of the films were assessed with Texture Analyzer TA.XT. Plus (Stable Microsystems, Godalming, UK), according to the previously optimized method [46]. For that purpose, 4 cm^2^ films were placed on the mucoadhesion ring A/Muc and moisturized with 250 µL of the SS pH 6.8. After 1 min of the thermostating process at temperature 37 ± 2 °C, upper probe with the buccal mucosa attached with a cyanoacrylate glue (round piece with r = 0.5 cm, S ~ 0.785 cm^2^) was lowered at a constant speed of 0.5 mm/s. Subsequently, the films were kept in contact with a model tissue for next 180 s under an initial contact force of 1 N and then two interacting surfaces were separated at post-test speed of 0.1 mm/s. Process parameters were chosen during the preliminary tests. While the maximum detachment force (F_MAX_) was recorded by Texture Exponent 32 software (version 5.0, Stable Microsystems, Godalming, UK), the work of adhesion (W_AD_) was calculated based on the area under force/distance curve as presented below,

W_AD_ = A × 0.1 × 1000,

where A is the area under force vs. distance curve. Multiplication by 0.1 concerns the conversion of time measurement to distance, while 1000 enables to express the parameter in µJ.

### 2.17. Antifungal Activity Test

The antifungal behavior of the films was determined against cultures *Candida albicans* ATCC^®^ 10231, *Candida krusei* ATCC^®^ 6528, and *Candida parapsilosis* ATCC^®^ 22019 according to the agar diffusion method of Clinical and Laboratory Standards Institute (CLSI), by using Sabouraud’s dextrose agar [47]. The inoculum of fungi was prepared with 0.9% sodium chloride and its target optical density of 5 × 10^6^ colony forming units per milliliter (CFU/mL) was determined by spectrophotometric method at 550 nm (Genesys 10S UV–Vis spectrophotometer, Thermo Scientific, Madison, WI, USA). After seeding of 100 μL of *Candida* species inoculum on a Petri dish, films pieces with diameter of 5 mm were carefully placed on the agar surface. Simultaneously, 100 μL of 1% (*w/v*) CLO dissolved in DMSO and 100 mg of the commercially available product with 1% (*w/w*) CLO were analyzed. The plates were subsequently incubated at 37.0 ± 0.1 °C either for 24 h (*C. albicans*, *C. krusei*) or 48 h (*C. parapsilosis*). After that time, the inhibition zones were measured [48]. For pure DMSO, no effect on the fungi proliferation was observed.

### 2.18. Cytotoxicity Tests

#### 2.18.1. Cells

Human gingival fibroblasts (ATCC^®^, PCS–201–018) were cultured with Dulbecco’s Modified Eagle’s Medium (DMEM) supplemented with 10% (*v/v*) fetal bovine serum (FBS), 100 U/mL penicillin, and 100 μg/mL streptomycin. Cells were seeded at a density of 10,000 cell/cm^2^ in 150 cm^2^ flasks (Corning^®^ cell culture flasks) and grown at 37 °C with 5% CO_2_. Cell viability was measured after each collection using optical microscopy with Trypan Blue staining. In all experiments the cell viability was >96%. Selection of the cell line was based on the previous investigations [49,50].

#### 2.18.2. Preparation of Samples

Films samples were placed in Eppendorf tubes and immersed in 2 mL of DMEM for 24 h extraction at room temperature [51]. After this time, the contents of the tubes were centrifuged at 4000 rpm for 10 min. The polymers concentration was 0.1%, 0.2%, 0.5%, and 1.0% (*w/v*). For testing, 50 µL of the obtained extracts was used.

#### 2.18.3. MTT Assay

After 24 h of incubation with formulations F1–F3 and the placebo films PF, the culturing medium was discarded and the cells were rinsed three times with phosphate-buffered saline (PBS, pH 7.4). Then, the cells were incubated for 20 min with MTT solution ((3–(4,5–dimethylthiazol–2–yl)–2,5–diphenyltetrazolium bromide), 5 mg/mL). Medium was removed from the wells, and the cells were dissolved in 200 μL of DMSO with 20 μL of Sorensen’s buffer (0.1 mol/L glycine with 0.1 mol/L NaCl equilibrated to pH 10.5). The absorbance was measured using spectrophotometer (Infinite M200 PRO Multimode Microplate Reader, Tecan, Männedorf, Switzerland) at 570 nm. Values were described as a percent of control [52].

#### 2.18.4. [3H]–Thymidine Incorporation (DNA Biosynthesis Assay)

Cells were seeded in 24-well plates at 1 × 10^5^ cells/well with 1 mL of growth medium. After 48 h to subconfluent cells, various concentrations of F1–F3 or PF films and 0.5 mCi of [3H]–thymidine were added. The incubation was continued for 24 h at 37 °C. Cells were rinsed three times with PBS, solubilized with 1 mL of 0.1 mol/L sodium hydroxide containing 1% SDS, then scintillation fluid Ultima Gold XR was added and incorporation of the tracer into DNA was measured in scintillation counter. Values were described as a percent of control [53].

#### 2.18.5. ROS Production Rate

The DCFH–DA (2′,7′–dichlorodihydrofluorescein diacetate) fluorescence method was used to detect the intracellular level of reactive oxygen species (ROS) [54,55]. Briefly, 2 × 10^4^ cells were grown in 96-well plates for 24 h, and then incubated with 20 mM DCFH–DA for 30 min in the dark. DCFH–DA is a non-fluorescent dye easily permeable into cells and then hydrolyzable by intracellular esterase to DCFH storage in the cells. At the end of DCFH–DA incubation, cells were washed with PBS. Cells were then treated with F1–F3 and PF films for 24 h. After washing, the formation of fluorescence dichlorofluoroscein, which is the oxidized product of DCFH–DA in the presence of several ROS (primarily hydroperoxide), was determined at 400/505 nm. Results were expressed as the fluorescence intensity.

#### 2.18.6. Real-Time-Glo^TM^ Annexin V Assay

The cells were seeded in 96-well plates at a density of 2000 cells/well, adhered overnight, then formulations F1–F3 and PF at polymers concentration of 0.2% and 0.5% were added and cultured at 37 °C, 5% CO_2_ for 24 h. To detect apoptosis and necrosis, Real-Time–Glo^TM^ Annexin V Apoptosis and Necrosis Assay Kit was utilized. This assay measures the exposure of phosphatidylserine (PS) on the outer leaflet of the cell membrane during the apoptotic process. H_2_O_2_ (50 mM) was used as the positive control.

### 2.19. Statistical Analysis

The quantitative variables were expressed as the mean ± standard deviation (SD) by MS Excel software. The measurements were considered significant at *p* < 0.05.

## 3. Results and Discussion

Design and development of the films were based on different physicochemical characteristics of the interacting polysaccharides. Due to pH-dependent solubility of CS, the polysaccharide layer was selected as that one responsible for prolonged delivery of CLO at the application site. Simultaneously, water-soluble and so easily disintegrating in the presence of the SS PC layer was recognized as adequate for providing initial dosage of CLO necessary for rapid saturating the affected area. According to that, different distribution of a drug in both polymers were applied. While in F1 and F2 formulations, CLO was incorporated in either polycation or polyanion, F3 films were analyzed with regard to controlled delivery of CLO from CS in the presence of drug-free PC layer acting as a barrier supporting prolonged in time antifungal effect. It should be emphasized that the addition of PEG 400 resulted in the visually homogenous drug distribution in the films matrices.

### 3.1. Evaluation of Films with Regard to Thickness, Weight Uniformity, Microenvironmental pH, Moisture, and Drug Content

All tested formulations were highly comparable in terms of their thickness and weight (Table 2). Films thickness was included in the range of 50 to 1000 µm regarded as optimal for buccal films [39]. Buffer capacity of the saliva solution was enough to maintain its physiological pH value in contact with the films what might eliminate the possible mucosa irritation effect caused by films application. In fact, the range of pH values recorded after 4-hour swelling of the films were 6.78–6.81. CLO content in all tested formulations was included in the range from 84.6% to 105.2% (Table 2). The highest values of the parameter were noted for F2 films (105.2 ± 8.1%), while F1 and F3 systems showed very similar values of CLO content (86.1 ± 6.6% and 84.6 ± 6.4%, respectively, for F1 and F3 films). It might be assumed that stronger electrostatic interactions between CS and PC in F2 might have resulted in higher entrapment efficiency of CLO in the interacting polymeric chains. These insights remain in correlation with low drug release rate from F2 (Section 3.7.) as well as the results obtained for CS/PC films by Maciel’s group [56]. Moisture content was ranged from 4.9 ± 1.1% to 7.9 ± 2.0% and so it was below the values obtained by Norcino et al. for CS/PC monolayer (blend) films [57]. Only negligible differences in the films’ humidity were probably without effect on their mechanical performance (Section 3.2.).

### 3.2. Physicomechanical Evaluation of Films

Due to the importance of optimal mechanical characteristics in films applicability, evaluation of the basic viscoelastic properties was performed. While the adequate mechanical strength is crucial for production, packaging or application processes, excessive elasticity of films might affect their further gluing, loss of a shape and impeded placing at the application site.

Despite different drug distribution in the films layers, no significant variations in the physicomechanical characteristics of the systems were observed (Table 3). In contrast to the results obtained by Centkowska et al. [40], incorporation of CLO did not markedly affect the mechanical resistance of F1–F3. F2 films with potentially the most intensive interpolymer complexation, as the in vitro release test might have suggested by prolonged in time release kinetics of CLO (Section 3.7.), showed the lowest mean value of elongation at break which might have resulted from the rigid PEC formation limiting elasticity of the films. Among all formulations tested, F2 films also performed the lowest values of folding endurance (Table 3). Even though, continuity of these films was preserved during 300 times folding, visible cracks were observed after on average 150 folds. F1, F2, and the placebo films PF were in turn sufficiently elastic to maintain their integrity despite exposure to the constant load. Similar to the previous scientific reports devoted to CS/PC films [58], low elasticity of the systems were highlighted despite addition of glycerol and PEG 400 as plasticizers. The effect of low mechanical performance upon PECs formation is also widely known in the literature [59,60] and so it is still challenging for the physical integrity and applicability of the films [39]. The percent elongation at break was comparable with, on average, 4–15% values noted for CS/PC monolayer (blend) films dedicated to vaginal delivery of fluconazole [32]. Similar results of the tensile strength were also noted.

### 3.3. Morphology of Films

**Figure 1:**
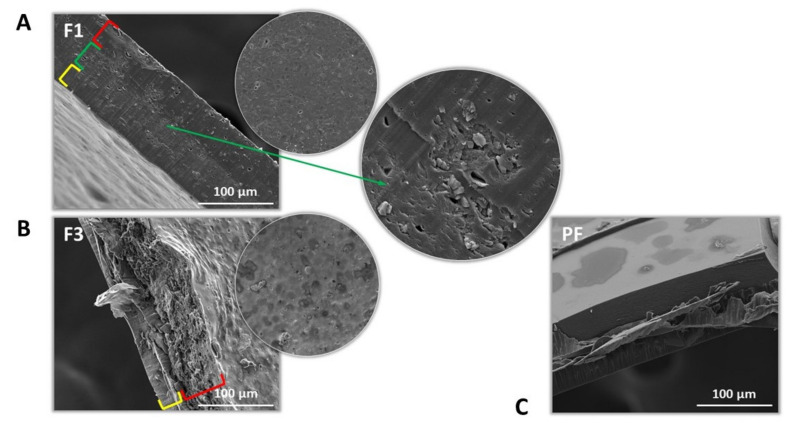
Representative cross-section and surface SEM images of the films F1 (**A**) and F3 (**B**), and cross-section picture of the placebo system (PF) (**C**) under 5000× magnification. The porous section of F1 films with CLO crystals was pointed. Visible layers of the systems formed were indicated with colors.

SEM analysis picturized the multilayer character of the films formed (Figure 1A–C). Both polymer’s layers were clearly displayed, however the layer of interacting CS and PC might have been indicated as the porous area of the films F1 with the accumulated particles of CLO (Figure 1A). The interpenetrating and ionically interacting polymeric chains of the casted CS through the preliminarily jellified PC layer might have resulted in the loose structure of the middle part of the films. Similar observations were made by Kononova et al. and Petrova et al. [17,61], where particular order of polymers mixing (polyanion as the first one) resulted in the porous interfacial layer of PEC formed. Morphological evaluation of the drug–deprived formulation PF showed the explicitly separated layers of CS and PC and most probably firm and brittle interlayer of a polycomplex (Figure 1C).

### 3.4. Thermal Behavior

**Figure 2:**
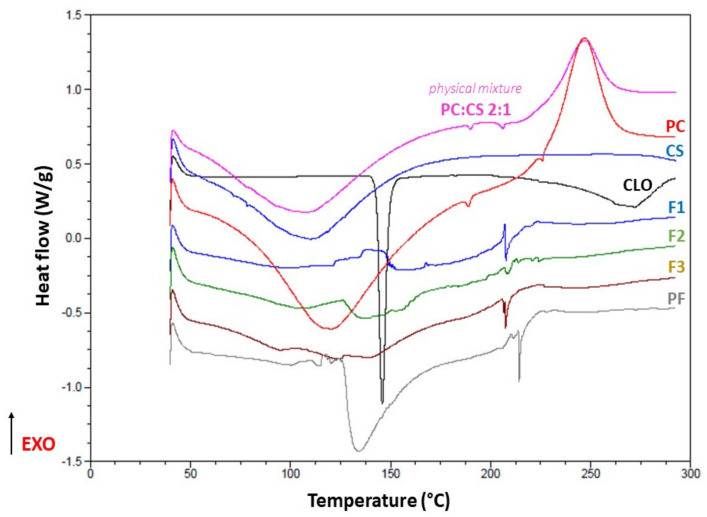
DSC thermograms for PC, CS, CLO, F1–F3, placebo films (PF), and the physical mixture of PC and CS at a weight ratio of 2:1.

The sharp endothermic peak at 147.59 °C corresponding to the crystal melting point of CLO [62] was recorded (Figure 2). The second one (272.01 °C) might have indicated possible contamination of the sample as, according to Garcia Ferreira et al. [63]., decomposition of the drug typically occurs above 340 °C. Similar to the previous scientific reports [64,65,66], the protective effect of the electrostatically interacting polymers on the active substance resulting from inclusion of the drug inside PECs can be observed as no peaks indicating the process of the drug decomposition were recorded. The absence of CLO melting point might have also resulted from undetectable drug amount in the films, crystallinity reduction or transition of CLO to its amorphous form as a result of the solubilizer (PEG 400) addition. The wide endothermic peaks in the range of 50 to 200 °C noted for CS, PC and physical mixture PC:CS 1:2 (*w/w*) most probably corresponded to the evaporation of the absorbed moisture. Exothermic peaks on PC and physical mixture thermograms (246.84 °C and 247.22 °C, respectively) were recognized as the points of thermal decomposition of PC [67]. As compared to the thermal behavior of F1–F3 and placebo films PF, ionic interactions between cationic CS and anionic PC resulted in the higher stability of PC against temperature since no peaks corresponding to the polymer disintegration process were noted. For the physical mixture of CS and PC, all peaks specific to the single polymers were recognized what might point out the absence of the interactions between the aforementioned components. We assume that the endothermic peak recorded for the placebo films at 133.77 °C might have resulted from the presence of PEG 400 not engaged in the solubilization of CLO but increasing water uptake by the films, thereby the enhanced moisture loss was recorded on the thermogram [68].

### 3.5. FTIR Analysis

The physical mixtures of PC:CS (2:1) and all designed films were analyzed by FTIR spectroscopy. The spectrum of the physical mixture was mainly dominated by PC signals (Figures S1 and S2). The characteristic bands appeared at 3360 cm^−1^, 1735 cm^−1^, 1605 cm^−1^, and 1440 cm^−1^, which can be correlated to –OH stretching vibration, C=O stretching of methylated carboxyl groups, and asymmetric or symmetric stretching vibrations of carboxylate groups, respectively [69].

Compared to the physical mixture of PC and CS, the spectrum of PF–CS side showed a decrease in intensity and a shift of the signal from the C=O group from 1735 cm^−1^ to 1726 cm^−1^, a shift of the symmetrical stretching vibration of the carboxyl group from 1440 cm^−1^ to 1452 cm^−1^, and the appearance of a broad band ranging from 1500 cm^−1^ to 1680 cm^−1^ (Figure S1). This indicates the formation of strong ionic interactions between amino groups of CS and carboxyl group of PC [56,58]. A similar trend was observed for CS side of F1–F3. As for the spectra of the other side of films, they highly resembled the physical mixture of PC and CS (Figure S2).

Additionally, FTIR spectra showed the presence of CLO in the films. In the spectra of both sides of the film F1, the presence of the drug can be confirmed based on the area at 800–600 cm^−1^ (Figure S2). However, the intensity of the signals differed depending on the side of the film, which indicated uneven distribution of CLO. It might be concluded that suspending of CLO particles as well as the mobility of CS chains during PC interpenetration might have influenced enhanced deposition of the drug in firstly casted PC layer. The presence of CLO was also found in the spectra of both sides of the film F2. In contrast, in the spectra of F3, CLO was only present on one side of the film.

### 3.6. Zeta Potential

Measurements of the surface charge of CLO particles aimed to recognize the impact of pH on CLO performance with particular attention given to its behavior against electrostatic interactions between positively charged CS and negatively charged PC. Considering high susceptibility of PECs to different internal and external factors, such as the charge of the incorporated active molecules, we wanted to verify any possible impact of CLO on ionically interacting polymers since there are scientific reports indicating correlation between ionization degree of the antifungal and pH conditions [70].

While particles dispersed in water were characterized with a charge of −43.2 ± 1.4 mV directly after suspending, the presence of ions in the SS resulted in significantly less negative values of the parameter (Table 4). Zeta potential values of the drug dispersed in the SS with pH 6.8 were −11.2 ± 0.6 mV, whereas acidifying the medium to pH 4.8 resulted in approximately three times higher values of the zeta potential (−4.1 ± 0.6 mV) what might have pointed out the enhanced CLO protonation with potential effect on the PEC structure [71]. We assume that upon decreasing the pH value, more protonated CLO might have disorganized the ionically interacting CS and PC chains. Except for CLO dispersed in the SS pH 6.8, reduction of the zeta potential values was recorded which might have arisen from the enhanced drug solubility over time. Particularly large differences in the particles charge were observed for the aqueous suspension. Nevertheless, the effect of CLO protonation upon pH decline was preserved, whereas reduction of the negative charge from −14.2 ± 1.1 mV to −2.7 ± 0.6 mV was recorded after 24 h.

### 3.7. In Vitro Release of CLO

**Figure 3:**
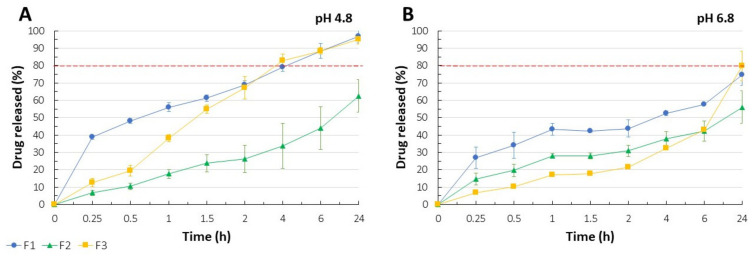
CLO release profiles noted for F1–F3 films in the SS at pH 4.8 (**A**) and pH 6.8 (**B**) at 37.0 ± 0.5 °C (mean ± SD, n = 3).

Acidifying the medium resulted in the accelerated release of CLO, except for F2 formulation which release profile was only slightly affected by pH change (Figure 3A–B). Only for F2, 80% of dosage was not released during 24-hour study regardless of the medium used. F1 and F3 films performed pH-responsive release kinetics. While at pH 6.8 80% of CLO was recorded at “24 h” time point, acidifying the saliva solution allowed for similar drug release after just 4 h. It was noticed that CLO incorporated into PC layer was responsible for burst release effect observed for F1 films (~40%) versus F3 with above 12% release after 15 min of the analysis at pH 4.8. Given the physicochemical characteristics of CS (pKa 6.2–7.0) and its better solubility in an acidic environment, the impact of more rapid disintegration of CS layer, as a result of the enhanced repulsion of the protonated amino groups, on the release profiles of F1 and F3 films could be indicated. With regard to F2 formulation, high proportion of ionically interacting polymers chains resistant to pH variations might have been the reason of the highly prolonged CLO delivery (~56–62% after 24 h) just as much as the negligible amount of a drug adsorbed at the films surface responsible for its rapid release, which according to the specificity of buccal drug administration, might be not applicable for the buccal mucosa. Based on the drug release profiles presented in Figure 3, F1 films might be regarded as the most promising for modified release of CLO. The observed burst release effect can be responsible for an initial dose and saturating the affected area with the antifungal while prolonged in time delivery of CLO is crucial for maintaining the minimal inhibitory concentration of the drug which is mandatory for the pharmacological effect. It might be concluded that differences in the polymers solubility and in the strength of CS–PC ionic interactions highly affected release kinetics of the systems formed. While relatively fast disintegrating PC layer was responsible for an initial dose of a drug, gradual erosion of PECs and not complexed CS most probably affected the prolonged release of CLO. Considering the chemical character of CLO, its enhanced protonation at pH 4.8 as compared to pH 6.8 might have resulted in the increased water solubility and more rapid drug release as presented in Figure 3 [71,72]. The above assumptions were confirmed in the zeta potential analysis of CLO suspensions (Section 3.6.). Moreover, repulsion forces between positively charged CS and CLO might have disorganized the films structure with subsequent higher drug dissolution rate from the more porous polymeric matrix. It should be highlighted that sensitivity of CS/PC PECs to pH variations has already been the object of studies conducted by Maciel et al. [56] and it should be carefully considered with regard to the potential film applicability. Because of the novelty of PEC-based multilayer films prepared with the solvent casting method, the obtained results might point out the potential of those multicompartment systems in controlled delivery of either systemic or local active substances through various routes of drug application.

### 3.8. Swelling Performance

It is commonly regarded that the degree of polymers hydration is crucial for their mucoadhesive performance or the rate of drug release since it affects the process of polymeric chains mobility, i.e., the ability of relaxation and interpenetration.

**Figure 4:**
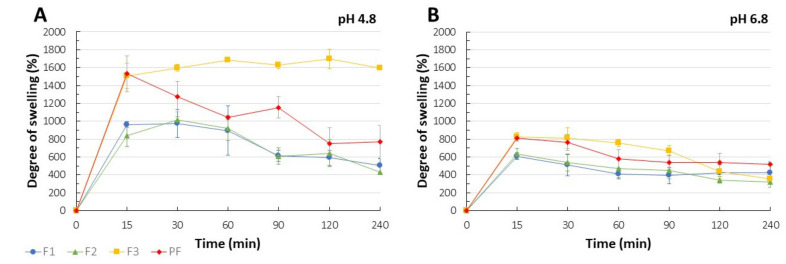
Swelling behavior of F1–F3 and placebo films (PF) in the SS with pH 4.8 (**A**) and pH 6.8 (**B**) at 37.0 ± 0.5 °C presented by using the degree of swelling (mean ± SD, n = 3).

Similar to the in vitro release study, pH values significantly affected the swelling performance of the systems formed (Figure 4A,B). F3 films performed the highest degree of swelling which was approximately two-fold higher at pH 4.8 as compared to pH 6.8. Gradual erosion and loss of the swollen films through the pores of the basket resulted in the progressively decreasing degree of swelling. Among all analyzed samples, compensation of the films erosion by swelling process was observed for F3 at pH 4.8, where only negligible weight fluctuations were noted during 4-hour test. Despite significant differences in CLO release profiles (Figure 3), similar swelling performance was observed for F1 and F2 films. Although the initial medium uptake (after 15 min) by the placebo films PF was comparable with F3 formulation and might have arisen from the uncontrolled swelling of the drug-deprived PC layer, intensive disintegration of the placebo films resulted in the degree of swelling after 4 h comparable to F1 and F2. We assume that the presence of “free” PEG 400 not involved in the solubilization process might have enhanced the hydration and erosion of CS and PC matrices as previously observed [73]. Except for F3 formulation, incorporation of CLO restricted the ability of media uptake. As mentioned above, the swelling performance observed for F3 might have arisen from the presence of drug-deprived layer of PC responsible for significant saliva uptake. While progressive erosion of the water-soluble polymer most probably resulted in the films loss, the increased swelling of CS layer at pH 4.8 might have been enough to compensate any disintegration processes. Nevertheless, high swelling degree cannot be recognized as the parameter pointing out poor interpolymer complexation. In fact, CS/casein PECs films developed by Pilicheva’s group [74] were characterized by significant water uptake capacity despite electrostatic interactions confirmed in FTIR assay. HMW CS is widely known for its long molecular chains with easily available hydroxyl groups able to stabilize ionic interactions with polyanions as well as to support water molecules entrance by hydrogen bonding [75].

### 3.9. Susceptibility of the Films to Erosion

The values of percentage erosion were comparable after 15 min and 60 min at pH 6.8 (Table 5). On the contrary, the films eroded in time after contact with the acidified SS and, surprisingly, F2 and F3 have undergone significantly slower erosion process as compared to pH 6.8. Considering different behavior of CS and PC in aqueous solutions, it can be assumed that not complexed and water soluble PC layer was quickly disintegrating while CS was mainly responsible for swelling of the remaining matrix. This different performance was predominantly observed at pH 4.8 where swelling of the films resulted in negligible increase of their size. Nevertheless, slower erosion under these conditions might point out the protecting effect of the swelling process on the films integrity. Variable outcomes recorded for F1 and F2 films after 15-minute contact with SS pH 4.8 presumably resulted from methodological obstacles in removing highly swollen and so very delicate films from the medium for drying. Relatively repeatable results noted for F3 and the placebo films PF can be related to their decreased disintegration noted in the swelling and disintegration test after 15 min (Section 3.8.). As it is difficult to correlate pH-dependent variations in CLO release kinetics with the outcomes of the erosion test after 60 min, we suspect significant impact of acidic pH on the enhanced CLO solubility. In addition, the attendance of insoluble CS/PC PEC in the prolonged delivery of CLO might be correlated with both in vitro dissolution and erosion test.

### 3.10. Mucoadhesive Properties

**Figure 5:**
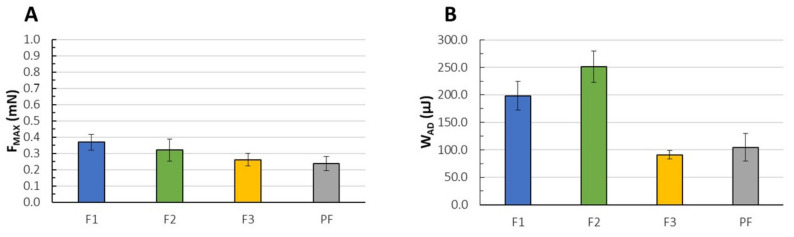
The maximum detachment force (F_MAX_) (**A**) and the work of adhesion (W_AD_) (**B**) recorded for F1–F3 and the placebo films (PF) (mean ± SD, n = 3, *p* < 0.05).

Considering the potential effect of the freezing process on the buccal mucosa integrity, we tried to minimize the effect of storage conditions on the measurement’s variations according to the previous attempts performed by our research group [46,48]. For that purpose, the buccal mucosa was carefully selected in order to eliminate potential differentiations arising from inhomogeneity of the mucosal surface. F3 and the placebo films PF performed significantly lower values of W_AD_ as compared to F1 and F2. Nevertheless, the values of F_MAX_ were very similar for all systems formed (Figure 5A,B). Substantial variations were observed only between F1 and both F3 and PF. We assume that swelling behavior might have affected the parameter of F_MAX_ and W_AD_. In fact, the absorption of water molecules in the highly swollen placebo and F3 films might have hampered interactions between the polymers and sialic acid of mucin. Even though the swelling ability is widely regarded as crucial for the mucoadhesiveness behavior since it increases mobility of polymeric chains and then access to the hydrophilic groups involved in the polymer–mucin interactions, detachment of the applied systems as a result of overhydration reduces the cohesion and mucoadhesion effect [76,77], what might have been observed in case of PF and F3 films. In response to the results obtained by Pilicheva et al. [74], a potentially dense and compact structure of F2 films (as dissolution rate might have suggested, Figure 3) cannot be treated as an obstacle in physical interpenetration of polymeric chains with mucin, instead both ionic and physical aspects of polymer–mucin interactions should be regarded as the authors emphasize.

### 3.11. Antifungal Activity

**Figure 6:**
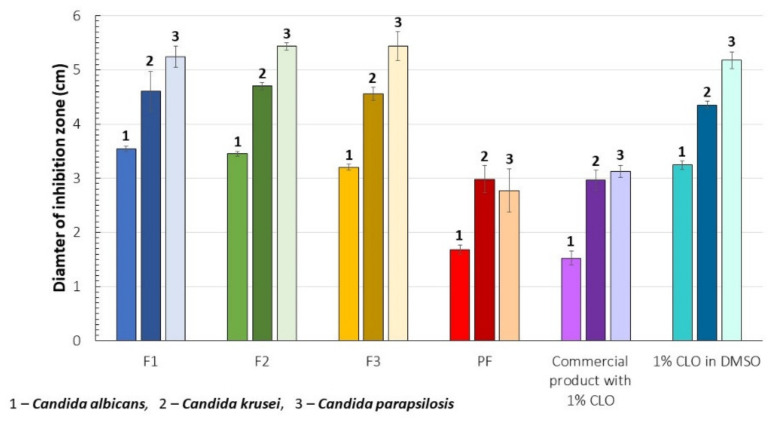
Antifungal activity of the films against *Candida albicans*, *C. krusei*, and *C. parapsilosis*. Diameters of the inhibitions zones observed for F1–F3 and the placebo films (PF) were presented with reference to the commercially available product with 1% CLO and 1% CLO dissolved in DMSO (*p* < 0.05).

No significant differences in the inhibition zones diameters were observed for the analyzed formulations (Figure 6). Nevertheless, the antifungal effect of CLO was increased by CS. In fact, the area of *Candida* sp. inhibition zones measured for the placebo films (PF) was comparable with the antifungal activity of 1% (*w/w*) CLO product used as a reference. The modulation effect of CS on the antimycotic behavior of CLO can be regarded as particularly important for lower risk of emergence of resistant *Candida* sp., mainly as a result of drug dose reduction or the modified mechanism of an antifungal action [78]. For all tested formulations, including the control samples, antifungal activity of CLO and CS was the weakest against *C. albicans*.

### 3.12. Cytotoxicity

**Figure 7:**
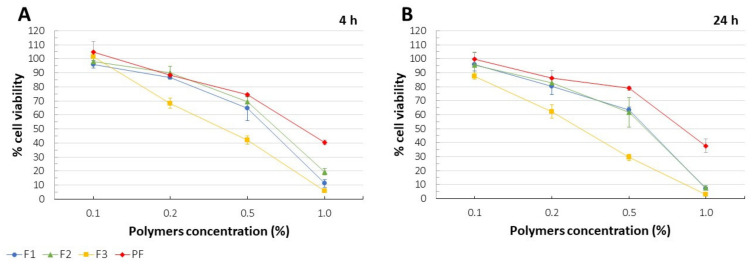
% cell viability determined in MTT assay after 4 h (**A**) and 24 h (**B**) for F1–F3 and placebo films (PF) at different polymers concentrations (mean ± SD, n = 4, *p* < 0.05).

For F1 and F2 films, no significant differences in MTT assay after 4 h (Figure 7A) and 24 h (Figure 7B) were recorded. While viability at the polymer concentration of 0.1% and 0.2% (*w/v*) were included in the range of 86.8 to 98.0% after 4h and 80.3–97.0% after 24 h, 1% concentration of the polymers resulted in significant cytotoxicity (cell viability below 20% after 4 h and below 10% after 24 h). As compared to the placebo films PF, variation in the cell viability was noted for the drug-loaded formulations with 1% polymers concentration what might point out potential toxic effect of CLO on mitochondrial function. F3 films performed the lowest values of cell viability within the range of 0.2 to 1.0%. We suspect that the presence of freely soluble in the medium PC layer without CLO might have resulted in the increased content of PC molecules in the extract. The enhanced polymer concentration observed for F3 formulation could be the reason of the observed cytotoxicity. Simultaneously, the protective effect of PECs on cells might have been indicated. In fact, F3 films with low interpolymer complexation and high attendance of free (positively or negatively charged) polyelectrolytes could be regarded as potentially toxic at concentration above or equal 0.2%, while strong electrostatic interactions between CS and PC in F1, F2, and PF formulations, and neutralized structures of PECs formation might have resulted in the enhanced biocompatibility of the systems. Incorporation of CLO only in the CS layer responsible for interpenetration and further interactions of the oppositely charged polymeric chains could result in low complexation of PC in F3 films. MTT test did not indicate any significant time-dependent cytotoxicity. Considering the results obtained in this cytotoxicity assay, the biocompatibility of the formulations F1–F3 and PF with human gingival fibroblasts up to 0.2% can be indicated, as over 80% cell viability was performed for the aforementioned concentrations.

**Figure 8:**
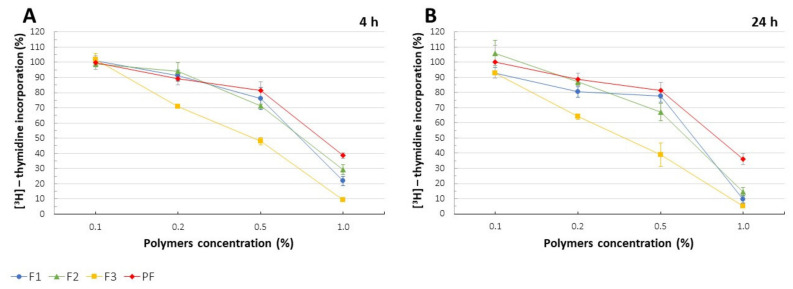
[3H]-thymidine incorporation (%) recorded for F1–F3 and placebo films (PF) after 4h (**A**) and 24 h (**B**) (mean ± SD, n = 4, *p* < 0.05).

DNA biosynthesis assay determines the incorporation of 3H-thymidine—a radioactive DNA precursor—into new strands of DNA during mitotic cell division. The results presented as percent 3H-thymidine incorporation when compared to the untreated cells were in high correlation with MTT assay (Figure 8A,B). While cell proliferation was not significantly affected by CLO addition, F3 films noticeably reduced 3H-thymidine incorporation which remained in accordance with the observations made in MTT test (see above).

**Figure 9:**
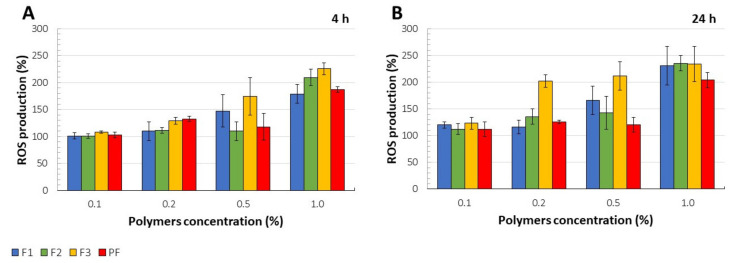
ROS production (%) for F1–F3 and placebo films (PF) after 4h (**A**) and 24 h (**B**) (mean ± SD, n = 4, *p* < 0.05).

ROS and specifically mitochondria-produced ROS are widely considered as factors affecting signaling cascades which regulate cell functions. It is commonly known that ROS production is inherent to mitochondrial oxidative metabolism, thereby methods of ROS determination have been applied for cytotoxicity evaluation [79]. Significantly higher ROS production as compared to the untreated cells was observed for F3 films at polymers concentration of 0.2–1.0%, especially after 24 h cell treatment (Figure 9A,B). For 1% concentration, about two-fold higher intensity of fluorescence and the corresponding ROS synthesis was recorded as compared to the control.

**Figure 10:**
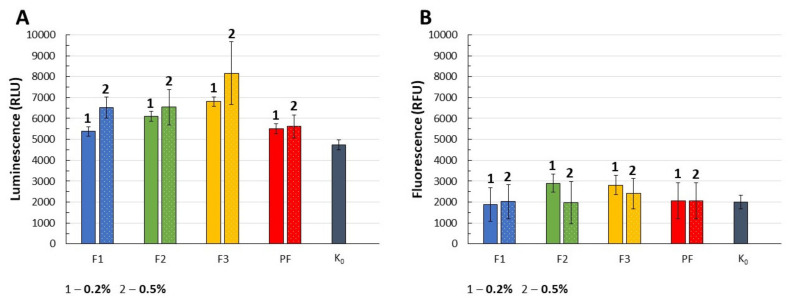
Intensity of luminescence signal as a sign of apoptosis (**A**) and fluorescence signal as a sign of necrosis (**B**) recorded for F1–F3 and placebo films (PF) at concentrations of 0.2% and 0.5% with reference to the negative control K_0_ (untreated cells). For the positive control (50 mM H_2_O_2_), luminescence was 964542.28 ± 32319.18 RLU and fluorescence–35750.39 ± 2510.64 RFU (mean ± SD, n = 5, *p* < 0.05).

The Real-Time–Glo™ Annexin V Apoptosis and Necrosis Assay measures the exposure of PS outside the cell membrane during the apoptotic process which results in structural changes of the cell membrane with subsequent translocation of PS from the cell to the extracellular side. By using near-equimolar ratios of two annexin V fusion proteins with high affinity for the anionic PS, the externalization of the aforementioned phospholipid might be detected. In the assay, annexin V binding can be determined with a simple luminescence signal, while necrosis, with a fluorescence signal. Higher intensity of luminescence signals was observed for all analyzed formulations comparing to the negative control (K_0_), what might point out the enhanced risk of cells apoptosis upon contact with formulations at the applied polymers concentrations (Figure 10A). Due to the differences in annexin V binding between F2–F3 and placebo films PF, the effect of CLO on the increased apoptotic processes might be recognized. No significant increase of fluorescence signals eliminated the potential necrotic effect induced by the films (Figure 10B).

## 4. Conclusions

Applicability of the solvent evaporation technique in layer-by-layer polymers deposition and films formation was confirmed. Multilayer character of the buccal systems with different distribution of CLO determined their drug release behavior. pH conditions highly affected swelling performance and disintegration rate of the interacting polymers what resulted in pH-dependent release kinetics. The satisfactory safety profile and improved by CS antifungal activity of CLO indicated possible utilization of the films in the oral candidiasis treatment. We believe that the potential of simply prepared multilayer films will be recognized as useful for the development of prolonged drug carriers intended for not only buccal but generally mucosal delivery of drugs. Since combination of CS and PC was found beneficial for the films preparation, further exploration of the possibilities which CS/PC PECs might offer in the technology of novel drug delivery systems is the purpose of our further research.

## Figures and Tables

**Table 1:** Percentage distribution of CLO in the films F1–F3.

Formulation	CLO Content (%)	
	PC Layer	CS Layer
F1	50.0	50.0
F2 ^1^	33.3	66.7
F3 ^1^	0.0	100.0

PC—pectin, CS—chitosan, CLO—clotrimazole, ^1^ Due to the limited solubility of CS, CS layer was proposed as a gradually eroded reservoir with higher drug content.

**Table 2:** Films characteristics: thickness, weight, moisture, and drug content (mean ± SD, n = 3).

Formulations	Thickness (µm)	Weight Uniformity (mg)	Moisture Content (%)	CLO Content (%)
F1	127.0 ± 14.8	30.8 ± 4.2	5.0 ± 1.1	86.1 ± 6.6
F2	147.0 ± 11.8	28.2 ± 2.1	7.9 ± 2.0	105.2 ± 8.1
F3	128.9 ± 6.7	23.5 ± 1.8	4.9 ± 1.1	84.6 ± 6.4
PF	132.5 ± 16.0	23.9 ± 5.1	7.0 ± 3.6	not applicable

**Table 3:** Physicomechanical characteristics of the films: Young’s modulus, tear resistance, tensile strength, elongation at break (mean ± SD, n ≥ 6), and the values of folding endurance (n = 3).

Formulation	Young’s modulus (kPa)	Tear Resistance (N)	Tensile Strength (N/mm^2^)	Elongation at Break (%)	Folding Endurance
F1	35.9 ± 1.4	34.1 ± 4.2	14.3 ± 2.0	6.2 ± 1.3	>300
F2	33.7 ± 7.2	25.6 ± 9.8	7.0 ± 1.3	3.0 ± 1.5	>150
F3	26.1 ± 8.9	24.9 ± 1.0	9.5 ± 1.9	6.9 ± 1.8	>300
PF	35.3 ± 7.1	34.9 ± 7.1	11.5 ± 2.7	5.5 ± 1.1	>300

**Table 4:** Zeta potential of CLO suspensions after T_0_ and T_24h_ at 25 ± 1 °C (mean ± SD, n = 3).

CLO Suspension	Zeta Potential (mV)	
	T_0_	T_24h_
CLO in water	−43.2 ± 1.4	−21.1 ± 2.1
CLO in SS ^1^ pH 4.8	−4.1 ± 0.6	−2.7 ± 0.6
CLO in SS ^1^ pH 6.8	−11.2 ± 0.6	−14.2 ± 1.1

^1^ SS with addition of 0.5% (*w/v*) Tween 80 to increase solubility of CLO.

**Table 5:** Percentage of erosion of the films F1–F3 and the placebo films PF after 15 and 60 min at different pH conditions (mean ± SD, n = 3).

Formulation	Erosion (%)			
	15 Min		60 Min	
	pH 4.8	pH 6.8	pH 4.8	pH 6.8
F1	39.06 ± 19.24	56.06 ± 9.00	50.99 ± 8.59	48.4 ± 1.83
F2	24.1 ± 16.16	50.01 ± 0.43	34.09 ± 5.38	57.53 ± 0.04
F3	26.79 ± 5.40	50.03 ± 12.52	35.54 ± 3.94	51.34 ± 6.77
PF	26.12 ± 6.94	59.10 ± 1.87	43.27 ± 10.03	50.91 ± 9.64

## Data Availability

FTIR measurements performed in the study are available in Supplementary Materials.

## Supplementary Materials

The following are available online at https://www.mdpi.com/article/10.3390/pharmaceutics13101588/s1, Figure S1: FTIR spectra of CLO, PC, CS, physical mixture of PC and CS at the ratio of 2:1 (*w/w*), and the placebo films (PF) from both sides (PC side, CS side), Figure S2: FTIR spectra of CLO, PC, CS, physical mixture of PC and CS at the ratio of 2:1 (*w/w*), and F1 films from both sides (PC side, CS side).
