# Supplementary material for: Multilayer Films Based on Chitosan/Pectin Polyelectrolyte Complexes as Novel Platforms for Buccal Administration of Clotrimazole
Source: Pharmaceutics. 2021 Sep 30;13(10):1588. doi: 10.3390/pharmaceutics13101588 (PMC8538955; doi:10.3390/pharmaceutics13101588)
Supplement: Supplementary file 1 [file pharmaceutics-13-01588-s001.zip › pharmaceutics-1377350-supplementary.pdf]

pharmaceutics

MDPI

Supplementary Materials: Multilayer Films Based on Chitosan/Pectin Polyelectrolyte Complexes as Novel Platforms for Buccal Administration of Clotrimazole

Joanna Potaś, Emilia Szymańska, Magdalena Wróblewska, Izabela Kurowska, Mateusz Maciejczyk, Anna Basa, Eliza Wolska, Agnieszka Zofia Wilczewska and Katarzyna Winnicka

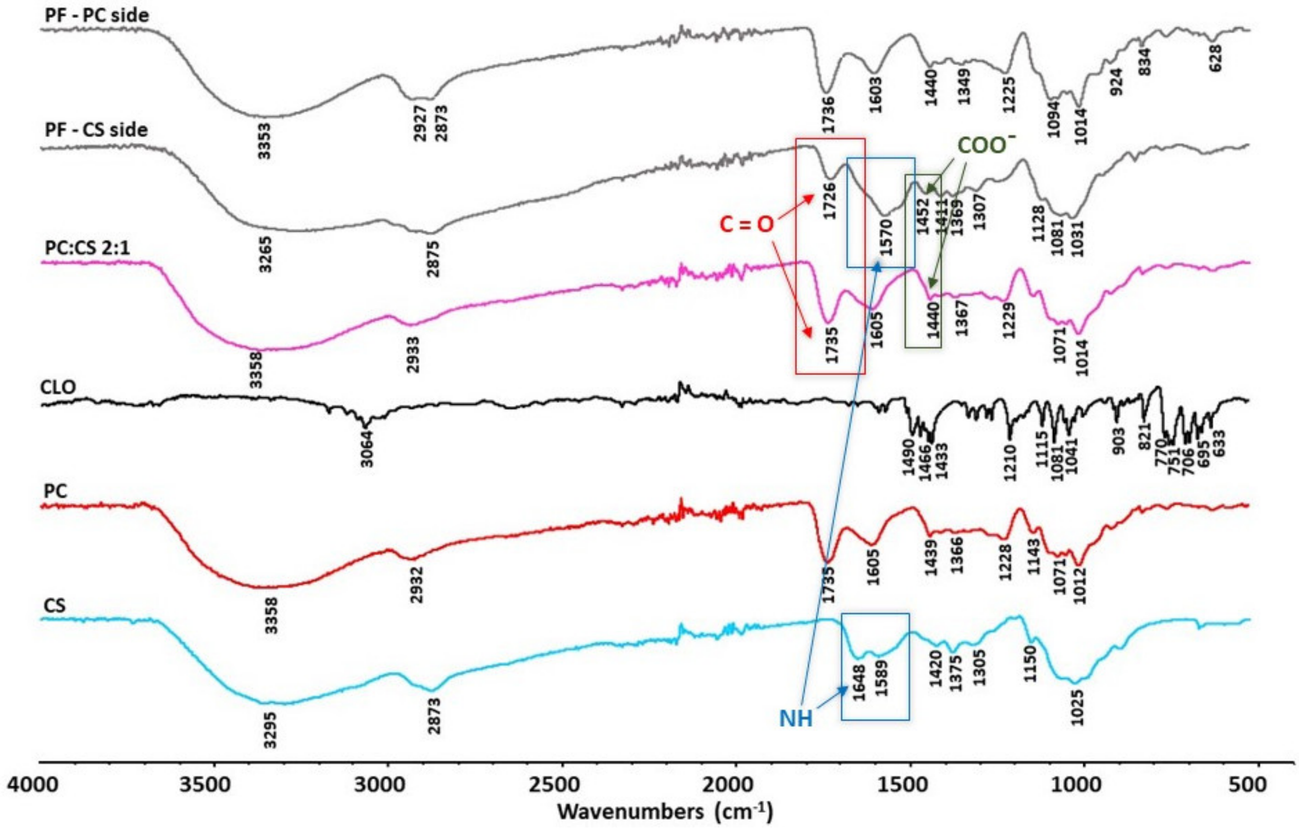

Figure S1. FTIR spectra of CLO, PC, CS, physical mixture of PC and CS at the ratio of 2:1 (*w/w*), and the placebo films (PF) from both sides (PC side, CS side).

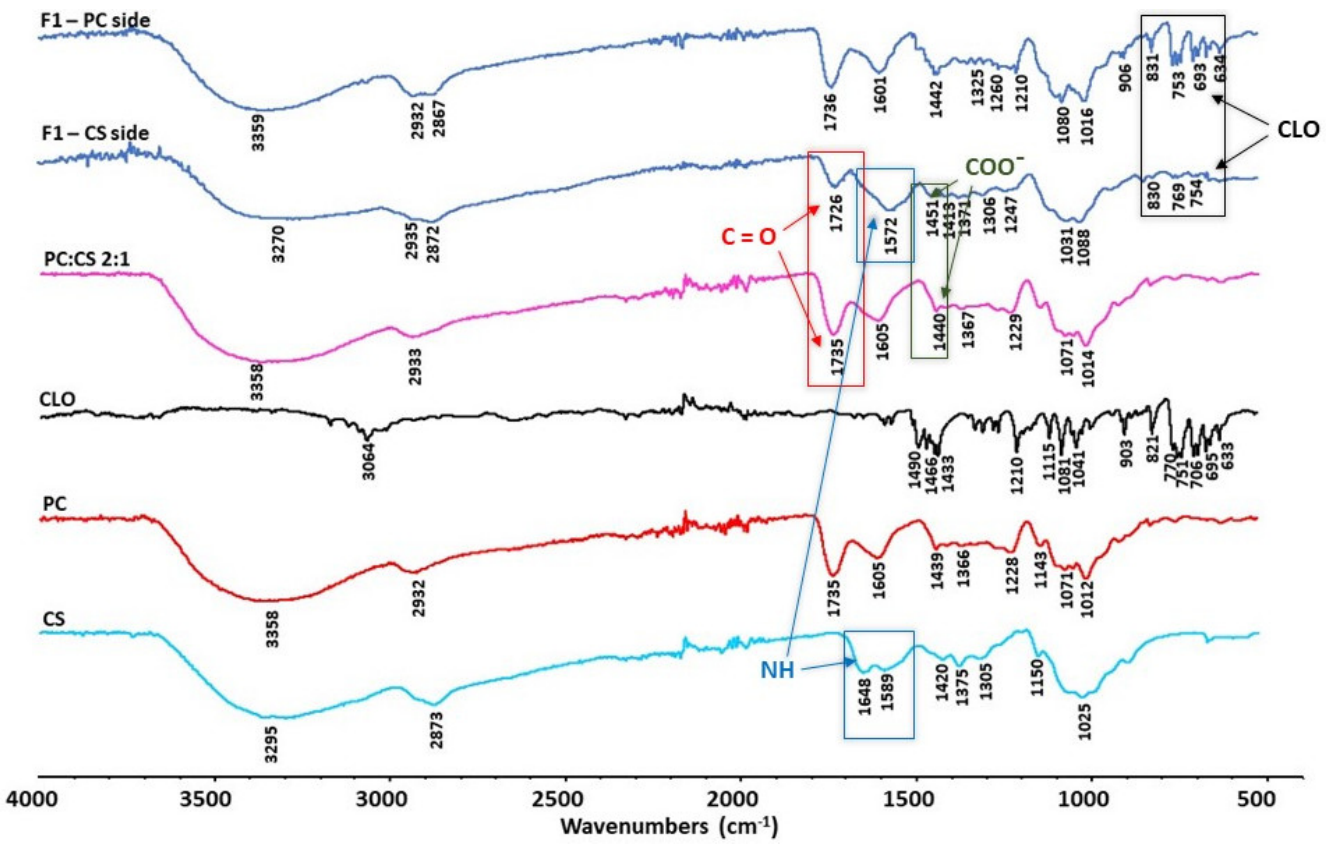

Figure S2. FTIR spectra of CLO, PC, CS, physical mixture of PC and CS at the ratio of 2:1 (*w*/*w*), and F1 films from both sides (PC side, CS side).
